# Gastric-protective and antioxidant effects of fermented *Pichia pastoris* residue hydrolyzed peptides on alcohol-treated mice

**DOI:** 10.1515/biol-2025-1257

**Published:** 2026-02-12

**Authors:** Yang Zhou, Yuxiang Huang, Shuaijun Guo, Chensi Wu, Xinru Shao, Chenlu Liu, Xinting Cui, Li Li, Hao Zang, Guangqing Xia

**Affiliations:** Tonghua Normal University, Tonghua, 134000, China; Key Laboratory of Evaluation and Application of Changbai Mountain Biological Gerplasm Resources of Jilin Province, Tonghua, 134000, China

**Keywords:** *Pichia pastoris* residue hydrolyzed peptides, ethanol-induced gastric injury, oxidative stress, anti-inflammatory, gastroprotection

## Abstract

Alcoholic gastric ulcer is a prevalent digestive disorder closely associated with oxidative stress and inflammatory damage, prompting growing interest in bioactive peptides as potential nutritional interventions. This study aimed to evaluate the nutritional composition, *in vitro* antioxidant activity, and gastroprotective effects of *Pichia pastoris* residue hydrolyzed peptides (PPRHP) in a mouse model of ethanol-induced gastric injury. Amino acid analysis revealed that PPRHP contains 17 amino acids with an essential-to-total amino acid ratio of 0.512. *In vitro* assays demonstrated strong radical (ABTS and hydroxyl) and hydrogen peroxide scavenging abilities, as well as copper ion chelation capacity, attributable to the reactive residues within the peptide sequences like histidine, tyrosine, and methionine. *In vivo*, pretreatment with PPRHP (200, 400, and 800 mg/kg) dose-dependently ameliorated ethanol-induced gastric damage, as evidenced by reduced ulcer area, submucosal edema, and leukocyte infiltration. Furthermore, PPRHP significantly reversed oxidative stress by elevating gastric superoxide dismutase and serum catalase levels while reducing gastric malondialdehyde and myeloperoxidase levels (*p* < 0.001), with the most pronounced effects observed at the medium dose of 400 mg/kg. These findings indicate that PPRHP, a peptide-rich and antioxidant substance, exerts significant protective effects against ethanol-induced gastric ulcers, likely through mechanisms involving free radical scavenging, metal ion chelation, inhibition of lipid peroxidation, and restoration of oxidative-antioxidative balance.

## 1 Introduction

Alcoholic gastric ulcer is a prevalent ethanol-related digestive disorder with high global incidence. Its pathogenesis is complex, involving oxidative stress imbalance, hyperactivation of inflammatory cascades, and impaired barrier function in the gastric mucosa [1], 2]. Chronic alcohol intake disrupts the mucus-bicarbonate barrier, the primary defense against acid and pepsin erosion. This disruption facilitates H^+^ back-diffusion, raising intracellular acid load and inducing cellular injury. Ethanol also uncouples the mitochondrial electron transport chain, leading to a burst of mitochondrial reactive oxygen species (ROS) [3]. Furthermore, ethanol metabolism perturbs systemic redox homeostasis by suppressing endogenous antioxidants such as superoxide dismutase (SOD) and catalase (CAT), thereby impairing the clearance of superoxide anions (O_2_^−^) and hydrogen peroxide (H_2_O_2_). Concurrent activation of myeloperoxidase (MPO) in neutrophils promotes excessive lipid peroxidation, elevating malondialdehyde (MDA) levels and establishing a self-amplifying cycle of oxidative injury [3], 4].

Oxidative stress further propagates damage through inflammatory signaling dysregulation, including aberrant ROS-NOX/Nrf2 pathways and hyperactivation of the TLR4/MyD88/NF-*κ*B axis. These mechanisms promote epithelial apoptosis and microcirculatory dysfunction, eroding mucosal integrity and increasing risks of ulcer recurrence and malignant progression [4], 5].

Current ulcer therapy relies heavily on pharmacologic agents such as proton pump inhibitors, which alleviate symptoms through acid suppression but do not directly repair oxidative damage or barrier deficits. Long-term proton pump inhibitor’ use is also associated with adverse effects, limiting their utility for chronic prevention in high-risk individuals [6]. In this context, nutritional interventions derived from natural sources – including herbs, fungi, and plants – have gained attention as complementary or alternative strategies for ulcer prevention and management.

Bioactive peptides, protein fragments released via enzymatic hydrolysis, represent a promising class of nutraceuticals. They are readily sourced, can be produced *in vitro* or *in vivo*, and exhibit diverse physiological activities such as lipid-lowering, antihypertensive, and anti-inflammatory effects [7]–9]. Their low cost, high safety, and efficient cellular absorption further underscore their potential in functional food and therapeutic applications [8], 9].

The yeast *Pichia pastoris* is widely used for recombinant protein production due to its high expression yield, eukaryotic post-translational modification capacity, and cost-effectiveness. It bridges the gap between prokaryotic and mammalian expression systems and supports large-scale production of vaccines, antibodies, and industrial enzymes [10]–12]. However, its fermentation residues – rich in proteins, polysaccharides, and other bioactive constituents – remain underexplored and are typically relegated to low-value uses such as feed or fertilizer [13], 14].

Peptide-based therapeutics are increasingly recognized for their specificity, biocompatibility, and low toxicity, holding particular promise for precision and personalized medicine [15], 16]. This study aims to evaluate the amino acid composition, *in vitro* antioxidant capacity, and *in vivo* gastroprotective efficacy of *P. pastoris* residue-hydrolyzed peptides (PPRHP) in a murine model of ethanol-induced gastric ulcer. To our knowledge, this work is the first to comprehensively assess the potential of PPRHP in ulcer prevention and treatment. Our findings support the high-value utilization of this industrial by-product and highlight PPRHP as a candidate natural agent for alleviating alcohol-induced gastric injury, likely through modulation of oxidative and inflammatory pathways.

## 2 Materials and methods

### 2.1 Materials

Twenty-four male Kunming mice (SPF grade, aged 5 weeks, weighing 28–32 g) were obtained from Liaoning Changsheng Biotechnology Co., Ltd. (animal license number: SCXK (Liao) 2020-0001). The mice were housed under a 12 h light-dark cycle with *ad libitum* access to food and water. Following a 3-day acclimation period, the experiments were initiated. *P. pastoris* residue was provided by Tonghua Anruite Biopharmaceutical Co., Ltd., and the hydrolyzed peptides derived from it (PPRHP) were supplied by the School of Life Sciences at Tonghua Normal University.

**Ethical approval:** The research related to animal use has been complied with all the relevant national regulations and institutional policies for the care and use of animals, and has been approved by the Institutional Animal Care and Use Committee of Tonghua Normal University (approval code: 2024039; approval date: June 18, 2024).

### 2.2 Reagents and chemicals

The following chemicals and reagents of analytical grade were purchased from Sinopharm Chemical Reagent Co., Ltd.: absolute ethanol, salicylic acid, ferrous sulfate heptahydrate (FeSO_4_·7H_2_O), phenol, phosphate buffer solution (PBS), 30 % hydrogen peroxide (H_2_O_2_), acetic acid, 2,2′-Azino-Bis(3-Ethylbenzothiazoline-6-Sulfonicacid) Diammonium Salt (ABTS), gallic acid, potassium persulfate, ethylenediaminetetraacetic acid disodium salt dihydrate (EDTANa_2_·2H_2_O), copper sulphate (CuSO_4_), pyrocatechol violet, sodium acetate, 4 % paraformaldehyde, bovine serum albumin, 6-hydroxy-2,5,7,8-tetramethylchroman-2-carboxylic acid (Trolox), Coomassie Brilliant Blue G-250, 4-aminoantipyrine, hydrochloric acid and phosphoric acid. Horseradish peroxidise (EC 1.11.1.7) was sourced from Sigma-Aldrich. All reagents and solvents used were analytical grade. Commercial assay kits for MPO and SOD were obtained from Elabscience. The BCA protein assay kit was sourced from Beyotime, while kits for MDA and CAT were procured from Nanjing Jiancheng Bioengineering Institute.

### 2.3 Methods

#### 2.3.1 Analysis of the amino acid composition of PPRHP

The amino acid composition of PPRHP was determined through acid hydrolysis and post-column derivatization [17]. Briefly, 50 mg of PPRHP was hydrolyzed with 10 mL of 6 mol/L HCl at 110 °C for 24 h under a nitrogen atmosphere. After cooling, the hydrolysate was filtered through quantitative filter paper into a 50 mL volumetric flask. The residue was washed gradiently with deionized water (3 × 5 mL), and the combined filtrate was diluted to volume. A 1 mL aliquot of this solution was dried using a vacuum deacidification instrument at 60 °C, reconstituted in 1 mL of pH 2.2 sodium citrate buffer, and filtered through a 0.22 μm organic membrane. Analysis was performed on a Hitachi L-8900 automated amino acid analyzer equipped with a Hitachi ion-exchange column (4.6 mm × 60 mm, 3 μm particle size) and a post-column ninhydrin derivatization system with dual-wavelength photometric detection at 570 nm and 440 nm. The system featured a dual-temperature control system (reaction column: 115 °C; separation column: 70 °C). Separation was achieved using a lithium citrate buffer at a flow rate of 240 μL/min.

#### 2.3.2 Determination of *in vitro* antioxidant activity

##### 2.3.2.1 Determination of antioxidant activity by ABTS assay

The ABTS radical scavenging assay was performed according to our previous method [18]. Briefly, 10 μL of the PPRHP solution was mixed with 190 μL of the prepared ABTS radical solution (3.7 mM). The mixture was incubated in the dark at room temperature for 20 min, after which the absorbance of the sample was recorded at 734 nm. Trolox was used as positive reference. The IC_50_ values were calculated and expressed as the mean ± standard deviation (SD) in μg/mL.

##### 2.3.2.2 Determination of antioxidant activity by hydroxyl radical assay

The hydroxyl radical scavenging assay was conducted following the method of Asiwe et al. [19] with slight modifications. In brief, 50 μL of FeSO_4_ (3 mM), 50 μL of the PPRHP solution, and 50 μL of H_2_O_2_ (3 mM) were combined and allowed to react for 10 min. Subsequently, 50 μL of salicylic acid (6 mM) was added to the mixture. After a 30 min reaction period, the absorbance was measured at 492 nm. Trolox was used as a positive control. The scavenging activity was quantified, and the IC_50_ values were calculated and expressed as the mean ± SD in μg/mL.

##### 2.3.2.3 Determination of antioxidant activity by H_2_O_2_ assay

The H_2_O_2_ scavenging assay was performed according to the method of Fernando et al. [20] with slight modifications. Briefly, 70 µL of phenol (12 mM), 20 µL of 4-aminoantipyrine (0.5 mM), 32 µL of H_2_O_2_ (0.7 mM), 8 µL of horseradish peroxidase (1 U/mL), and 70 µL of the PPRHP solution were mixed in a microplate. All reagents were prepared in PBS, 84 mM, pH 7.0. The absorbance was immediately measured at 504 nm, using a blank where the phenol solution was replaced with PBS as a reference. Gallic acid served as the positive control. The IC_50_ value, defined as the concentration required to scavenge 50 % of H_2_O_2_, was calculated and is expressed as the mean ± SD in µg/mL.

##### 2.3.2.4 Determination of antioxidant activity by copper chelating assay

The copper ion chelating activity was determined following the method of Anne et al. [21] with slight modifications. Briefly, 40 µL of the PPRHP solution was mixed with 140 µL of acetate-sodium acetate buffer (50 mM, pH 6.0) and 10 µL of CuSO_4_ solution (5 mM). The mixture was incubated for 30 min. Subsequently, 10 µL of pyrocatechol violet (4 mM) was added, followed by a further 30 min incubation. The absorbance was then measured at 632 nm. A blank, in which pyrocatechol violet was replaced with deionized water, was used as the reference. EDTANa_2_ served as the positive control. The IC_50_ value, representing the concentration required for 50 % chelation, was calculated and expressed as the mean ± SD in µg/mL.

#### 2.3.3 Animal experimental design and *in vivo* procedures

##### 2.3.3.1 Ethanol-induced gastric ulcer model and intervention

A total of 36 healthy SPF-grade mice were randomly divided into six groups (*n* = 6). Group I (GI, blank control) received 0.5 % carboxymethylcellulose sodium; Group II (GII, model) received 0.5 % carboxymethylcellulose sodium and was later challenged with anhydrous ethanol; Group III (GIII, positive control) was administered omeprazole at 30 mg/kg body weight (BW), a dose selected based on its well-documented efficacy as a proton pump inhibitor that irreversibly blocks H^+^/K^+^ ATPase in gastric parietal cells, thereby suppressing acid secretion and providing gastroprotection in ethanol-induced injury models [22]; Groups IV (GIV), V (GV), and VI (GVI) received PPRHP at 200, 400, and 800 mg/kg BW, respectively. All treatments were administered orally once daily for 13 consecutive days. Following a 24 h fast (with free access to water), 2 h after the final treatment, Group I received normal saline (0.1 mL/10 g BW), while Groups II–VI were administered anhydrous ethanol (0.1 mL/10 g BW) to induce acute gastric injury. No mortality occurred following ethanol administration. All mice were then anesthetized with pentobarbital sodium (50 mg/kg BW, intraperitoneal). Blood was collected from the abdominal aorta, allowed to clot for 1 h at room temperature, and centrifuged at 3,500 rpm for 10 min at 4 °C to obtain serum, which was stored at −80 °C. Gastric tissues were promptly excised, rinsed with ice-cold saline, blotted dry, and weighed. A 10 % homogenate was prepared in normal saline, centrifuged at 8,000 rpm for 10 min at 4 °C, and the supernatant was stored at −80 °C for subsequent analysis.

##### 2.3.3.2 Assessment of organ indices

Following euthanasia, the final body weight of each mouse was recorded. The liver, spleen, and kidneys were then carefully excised, and their wet weights were measured. The organ index (expressed as a percentage) for each tissue was calculated as follows:

$$\text{Organ index }\left(\%\right)=\left[\text{Organ weight }\left(g\right)/\text{Body weight }\left(g\right)\right]\times 100\%$$

##### 2.3.3.3 Histopathological examination

Immediately after euthanasia by cervical dislocation, the stomach was excised and incised along the greater curvature. The gastric contents were gently rinsed away with ice-cold saline, and the tissue was flattened on clean filter paper for macroscopic imaging. The severity of gastric lesions was evaluated according to the established scoring system of Ren et al. [23]. Briefly, localized congestion/redness, punctate hemorrhage, and erosion were each assigned one point, while linear erosions were assigned three points. The cumulative score for each stomach was used for statistical analysis. Lesions were further graded on a scale of 1–4: Grade 1 (small areas, <4 lesions), Grade 2 (4–8 small ulcers), Grade 3 (9–16 small ulcers or several larger ones), and Grade 4 (large confluent ulcers, >16 small ulcers, or near-perforation). The ulcer area percentage and the ulcer inhibition rate were calculated using formula (1) and formula (2), respectively:

(1) $$\text{Ulcer area }\left(\%\right)=\left(\text{Gastric ulcer area}/\text{Total gastric area}\right)\times 100\%$$

(2) $$\text{Ulcer inhibition rate }\left(\%\right)=\left(1-\left[\text{Treated group ulcer area }\left(\%\right)/\text{Model group ulcer area }\left(\%\right)\right]\right)\times 100\%$$

##### 2.3.3.4 Histological assessment

For histological examination, gastric tissue specimens from three randomly selected mice per group were processed routinely. Briefly, the tissues were fixed in 4 % paraformaldehyde, dehydrated through a graded ethanol series, cleared in xylene, and embedded in paraffin. The embedded blocks were sectioned at a thickness of 4 μm. Following deparaffinization and rehydration, the sections were stained with hematoxylin and eosin. Pathological changes in the gastric mucosa were then examined and recorded under a light microscope.

##### 2.3.3.5 Biochemical analyses

Oxidative stress parameters in both serum and gastric tissues were evaluated using commercial assay kits according to the manufacturers’ instructions. A 10 % (w/v) gastric tissue homogenate was prepared with pre-cooled saline at a 1:9 tissue-to-saline ratio and centrifuged to collect the supernatant. The levels of MPO, SOD, and MDA in the gastric tissue were determined using kits from Nanjing Jiancheng Bioengineering Institute. For serum analysis, blood samples were collected from the orbital plexus, allowed to clot for 1 h, and centrifuged at 3,000 rpm for 8 min at 4 °C to obtain serum. CAT activity was also measured using the corresponding Jiancheng assay kit.

### 2.4 Statistical analysis

The data are presented as the mean ± standard deviation. Statistical significance was determined by one-way analysis of variance followed by post-hoc tests (least significant difference and Duncan’s test). A *p*-value of less than 0.05 was considered statistically significant, while *p*-values less than 0.01 and 0.01 were considered highly significant and extremely significant, respectively.

## 3 Results

### 3.1 Analysis of the amino acid composition of PPRHP

As shown in Table 1, the amino acid composition analysis of PPRHP identified 17 amino acids with a total content of 78.356 mg/g, comprising seven essential amino acids (EAAs) and ten non-essential amino acids (NEAAs). According to the ideal amino acid pattern proposed by the World Health Organization and the Food and Agriculture Organization, a high-quality protein should possess an EAA-to-NEAA ratio above 0.6 and an EAA-to-total amino acid (TAA) ratio around 0.4. The measured EAA/TAA and EAA/NEAA ratios of PPRHP were 0.512 and 1.049, respectively, both of which exceed these standard reference values. This profile indicates that PPRHP is rich in essential amino acids and closely approximates the criteria of an ideal protein, supporting its nutritional suitability for further experimental investigation.

**Table 1:** The amino acid composition and content of PPRHP.

Kinds of amino acid	Content (mg/g)	Kinds of amino acid	Content (mg/g)
Asp	2.401	Phe	4.698
Thr	5.718	His	0.502
Ser	0.303	Lys	0.228
Glu	0.286	Arg	0.358
Gly	4.600	Pro	10.504
Ala	17.075	Cys-Cys	0.686
Val	7.265	EAA	40.121
Met	5.671	NEAA	38.235
Ile	6.169	TAA	78.356
Leu	10.372	EAA/TAA	0.512
Tyr	1.520	EAA/NEAA	1.049

### 3.2 Antioxidant activity *in vitro*

As shown in Table 2, PPRHP exhibited dose-dependent *in vitro* antioxidant activity, demonstrating significant scavenging capacity against ABTS radicals, hydroxyl radicals, and H_2_O_2_, as well as copper ion chelation ability. The half-maximal inhibitory concentration (IC_50_) values for each assay were determined and compared with standard antioxidants. PPRHP showed an IC_50_ of 151.4 ± 0.6 μg/mL in the ABTS assay, 962.9 ± 8.5 μg/mL against hydroxyl radicals, 2,631.2 ± 33.7 μg/mL for IC_50_ scavenging, and 347.4 ± 19.6 μg/mL for copper ion chelation. These values were higher than those of the corresponding positive controls: Trolox (ABTS: 3.8 ± 0.1 μg/mL; hydroxyl radical: 97.5 ± 1.4 μg/mL), gallic acid (IC_50_: 65.5 ± 1.5 μg/mL), and EDTANa_2_ (copper chelation: 28.4 ± 1.4 μg/mL), indicating that although PPRHP possesses notable antioxidant properties, its activity in these assays was lower than that of the reference compounds.

**Table 2:** Determination of *in vitro* antioxidant indexes of PPRHP.

Sample	ABTS (IC_50_, μg/mL)	Hydroxyl radicals (IC_50_, μg/mL)	H_2_O_2_ (IC_50_, μg/mL)	Copper chelating (IC_50_, μg/mL)
PPRHP	151.4 ± 0.6^b^	962.9 ± 8.5^b^	2,631.2 ± 33.7^b^	347.4 ± 19.6^b^
Trolox^c^	3.8 ± 0.1^a^	97.5 ± 1.4^a^	N.T.	N.T.
EDTANa_2_^c^	N.T.	N.T.	N.T.	28.4 ± 1.4^a^
Gallic acid^c^	N.T.	N.T.	65.5 ± 1.5^a^	N.T.

^a–b^Columns with different superscripts indicate a significant difference (*p* < 0.05). ^c^Used as a standard antioxidant; N.T. indicates no test.

### 3.3 Body weight changes in animal models

Body weight was monitored every two days to assess the potential systemic effects of PPRHP administration. As summarized in Table 3, no significant differences in body weight were observed between the treatment groups and the control group throughout the experimental period. Furthermore, all mice maintained normal appetite, spontaneous activity, and fur condition, with no signs of adverse effects. These findings collectively indicate that the administered doses of PPRHP were well-tolerated and within a safe range.

**Table 3:** Body weight changes in murine models.

Groups	Day 1’s weight (g)	Day 3’s weight (g)	Day 5’s weight (g)	Day 7’s weight (g)	Day 9’s weight (g)	Day 11’s weight (g)	Day 13’s weight (g)
GI	37.70 ± 0.94	39.70 ± 0.73	39.10 ± 0.97	39.57 ± 1.19	40.05 ± 1.07	40.67 ± 0.90	41.12 ± 1.07
GII	37.42 ± 1.27	38.97 ± 1.16	39.35 ± 1.69	39.60 ± 1.77	39.97 ± 1.52	40.23 ± 1.38	40.65 ± 1.82
GIII	37.37 ± 0.97	38.25 ± 1.20	38.82 ± 1.03	39.12 ± 1.22	39.87 ± 1.57	40.15 ± 1.61	40.37 ± 1.47
GIV	37.75 ± 1.45	38.62 ± 0.98	39.15 ± 0.97	39.62 ± 1.26	40.27 ± 1.11	40.07 ± 1.22	41.00 ± 1.24
GV	37.35 ± 1.75	39.60 ± 1.50	40.07 ± 1.45	40.82 ± 1.64	41.70 ± 1.38	41.15 ± 1.40	42.10 ± 1.50
GVI	37.37 ± 0.60	38.17 ± 0.39	38.62 ± 0.50	38.80 ± 0.66	39.12 ± 0.88	39.42 ± 1.09	39.72 ± 0.97

### 3.4 PPRHP’s effects on organ indices in ethanol-induced gastric ulcer mice

**Figure 1:**
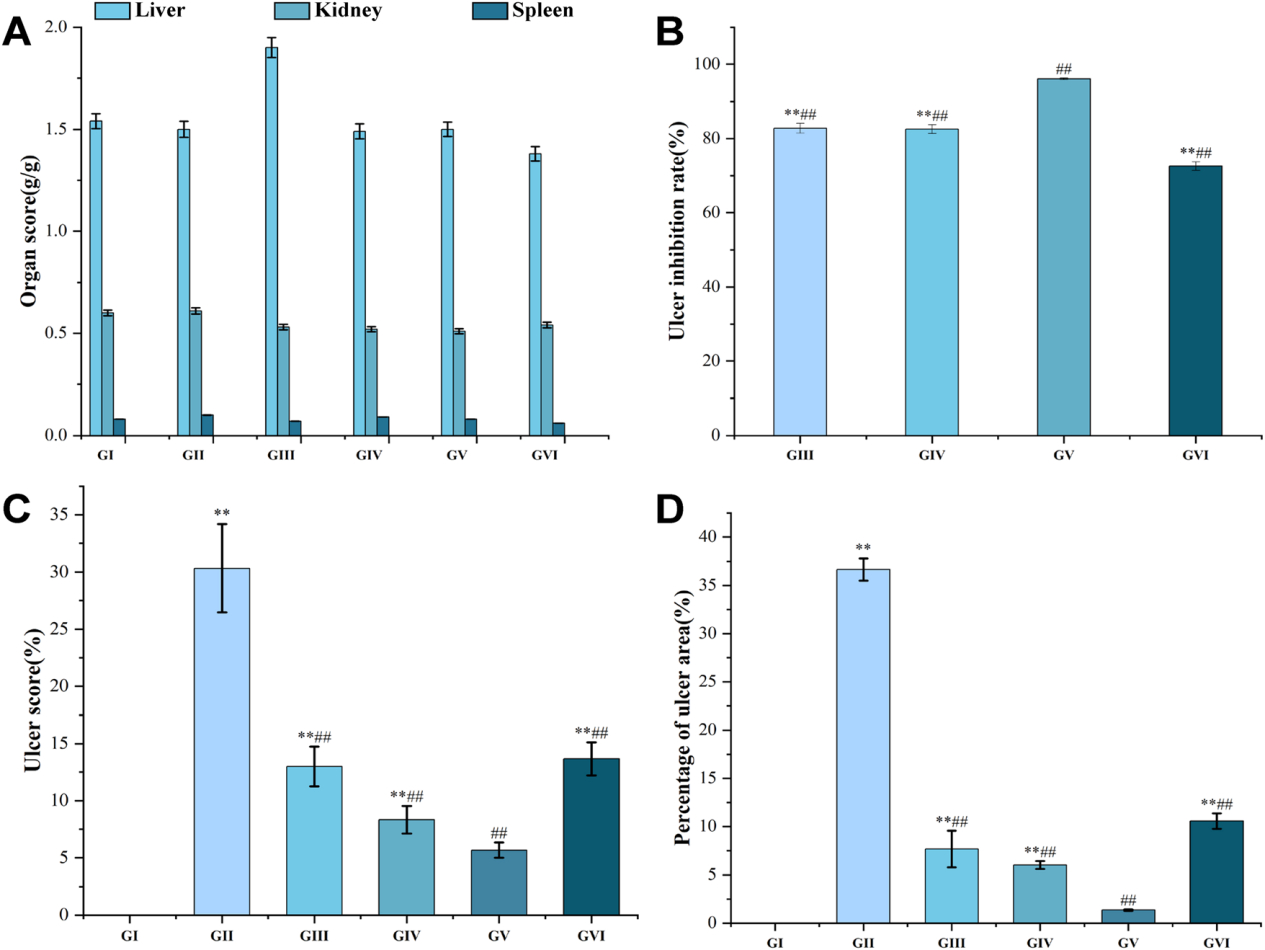
Key metrics of gastric ulcers in each group of mice ^**^represents *p* < 0.001 with the GI, ^##^represents *p* < 0.00*l* with the GII.

To evaluate the systemic safety of PPRHP, organ indices for the liver, spleen, and kidneys were calculated. As presented in Figure 1A, no statistically significant differences in organ indices were observed among any of the experimental groups. These results indicate that PPRHP administration did not induce notable organ weight changes, suggesting a favorable safety profile at the tested doses.

### 3.5 Effects of PPRHP on gastric tissue morphology in a murine model of ethanol-induced gastric ulcer

**Figure 2:**
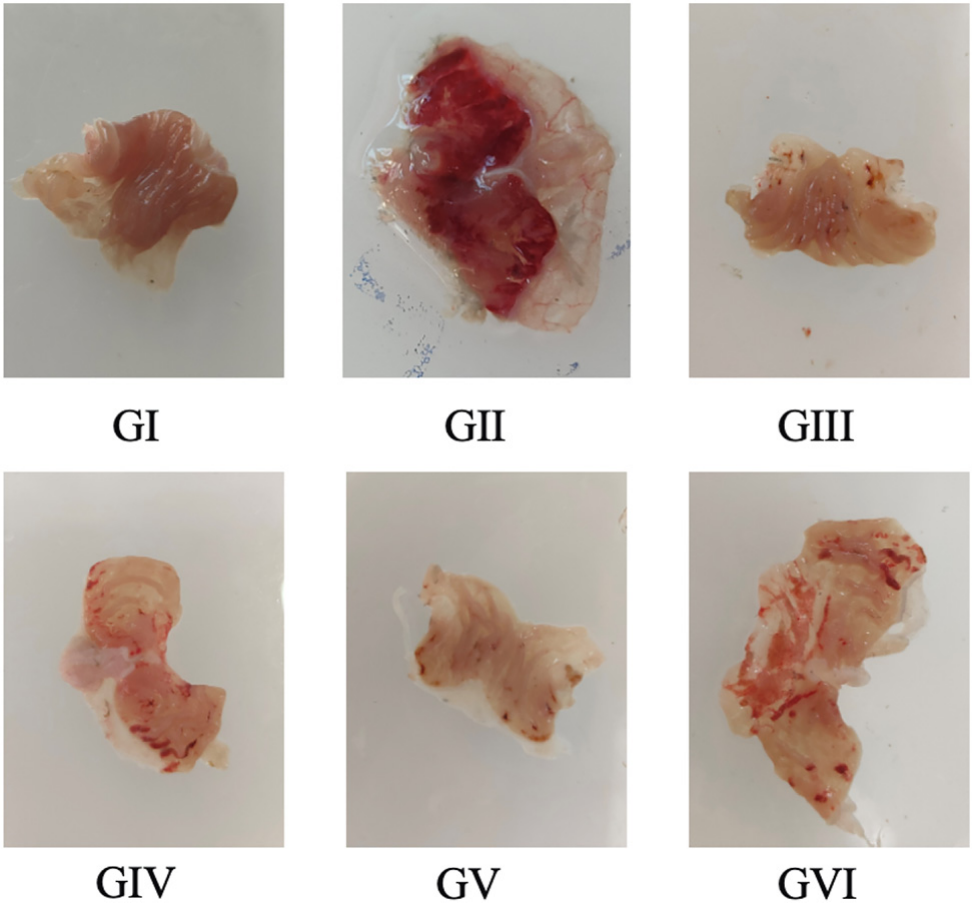
Representative macroscopic images of gastric tissues from each group.

Macroscopic assessment of gastric tissues was performed to evaluate the protective effect of PPRHP against ethanol-induced damage (Figure 2). In the normal control group, the gastric mucosa exhibited well-arranged folds, a light pink coloration, and an absence of hemorrhagic spots or other pathological alterations. In contrast, mice in the model group displayed severe gastric mucosal injury characterized by depressed centers and raised margins, irregular morphology, reddish erosions, prominent hemorrhagic spots, and tissue edema. Extensive hemorrhage and necrotic tissue were observed in the gastric fundus, accompanied by marked mucosal erosion and diffuse bleeding, resulting in a bright red appearance of the tissue. These findings confirm the successful induction of acute alcohol-induced gastric ulcers.

Pretreatment with omeprazole, the positive control, markedly preserved the integrity of the gastric wall and mucosa, with substantially fewer hemorrhagic spots and no evident congestion, edema, or erosion, supporting its therapeutic efficacy in this model. Similarly, all PPRHP-treated groups (low-, medium-, and high-dose) showed only minor hemorrhagic spots and significantly reduced lesion areas compared to the model group. Gastric surfaces in these groups were smoother, with no notable congestion, edema, or erosion. Overall, gastric morphology in the PPRHP-treated mice was significantly improved relative to the model group, with the most notable protection observed in the medium-dose (400 mg/kg) group.

Quantitative analyses further supported these observations (Figure 1A–D). Relative to the model group (GII), the low-dose PPRHP group showed significant improvements in ulcer score (8.33), ulcer inhibition rate (82.54 %), and ulcer area ratio (6.00 %) (*p* < 0.001). The medium-dose group exhibited even more pronounced effects, with an ulcer score of 5.66, an ulcer inhibition rate of 96.05 %, and an ulcer area ratio of 1.35 % (*p* < 0.001). The high-dose group also demonstrated significant amelioration compared to GII, with respective values of 13.66, 72.57 %, and 10.56 % (*p* < 0.001). Together, these results indicate that PPRHP exerts a dose-dependent ameliorative effect on ethanol-induced gastric ulcers, with the medium dose affording the most substantial protection.

### 3.6 Effects of PPRHP on gastric histopathology in a murine model of ethanol-induced gastric ulcer

**Figure 3:**
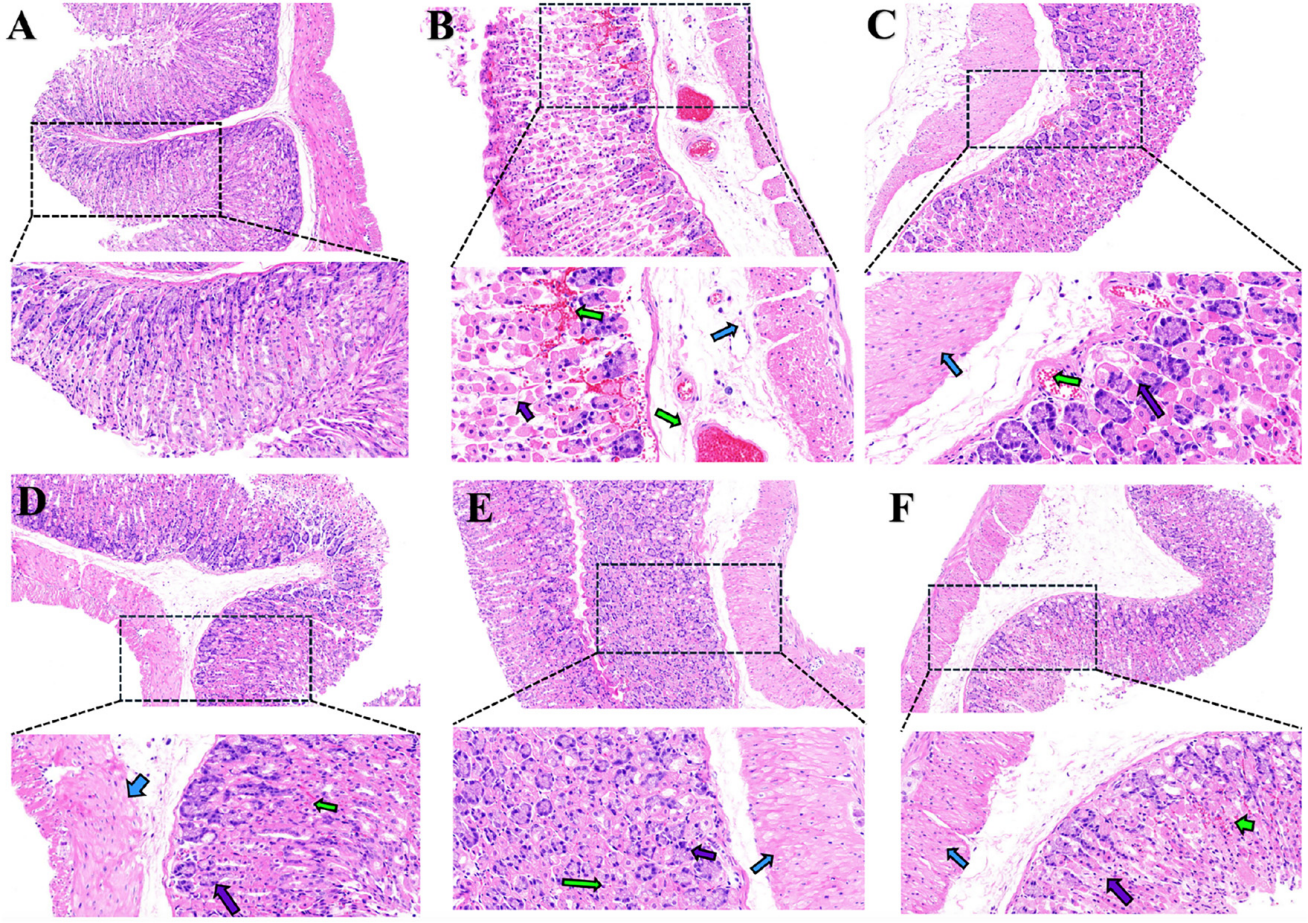
Representative histopathological photomicrographs of gastric tissue by group (A): blank control group (200×, 400× magnification); (B): model group (200×, 400× magnification); (C): omeprazole group (200×, 400× magnification); (D): low-dose group (200×, 400× magnification); (E): medium-dose group (200×, 400× magnification); (F): high-dose group (200×, 400× magnification). Purple arrow: gastric gland cells; green arrow: inflammatory cells; blue arrow: gastric muscle layer cells.

Histopathological examination of gastric tissues via hematoxylin and eosin staining was conducted to further characterize morphological alterations (Figure 3). In the blank control group, the gastric mucosa showed intact folds, orderly arranged epithelial and gastric gland cells, and preserved tissue integrity. The submucosa exhibited no edema, hemorrhage, significant vascular dilation, or inflammatory infiltration, and the gastric muscle layer displayed normal cellular morphology.

In contrast, the ethanol-induced model group exhibited severe disruption of gastric mucosal architecture, characterized by disorganized glandular structure, damaged gastric gland cells, irregular epithelial arrangement, and prominent inflammatory cell infiltration accompanied by extravasated red blood cells (indicated by green arrows). The submucosa showed marked vascular dilation with extensive inflammatory infiltration and hemorrhage (green arrows). The muscular layer also displayed severe injury, including abnormal cellular morphology, widened intercellular spaces, and disorganized alignment (blue arrows).

Treatment with omeprazole markedly attenuated these pathological changes, showing relatively intact mucosal architecture, only mild damage to gastric glands, and generally preserved tissue organization. Limited inflammatory cells and red blood cells were observed (green arrows), while the submucosa showed no notable vascular dilation or inflammatory infiltration. The gastric muscle layer appeared compact and nearly normal (blue arrows).

All PPRHP-treated groups exhibited improved gastric histology compared to the model group, with better-organized mucosal structure and reduced damage to gastric gland cells (purple arrows). The low-dose group showed mild inflammatory infiltration and scattered red blood cells (green arrows), whereas the high-dose group displayed further reduction in inflammatory cells and hemorrhage. Notably, the medium-dose group showed near-absence of inflammatory infiltration and red blood cells. No significant vascular dilation or submucosal inflammation was observed in any PPRHP-treated group, and the gastric muscle layer in all peptide-treated mice appeared compact and largely normal.

These histopathological findings demonstrate that PPRHP promotes structural recovery in ethanol-induced gastric ulcers, with the medium dose (400 mg/kg) conferring the most substantial reparative effect.

### 3.7 Gastric antioxidant protection of PPRHP

**Figure 4:**
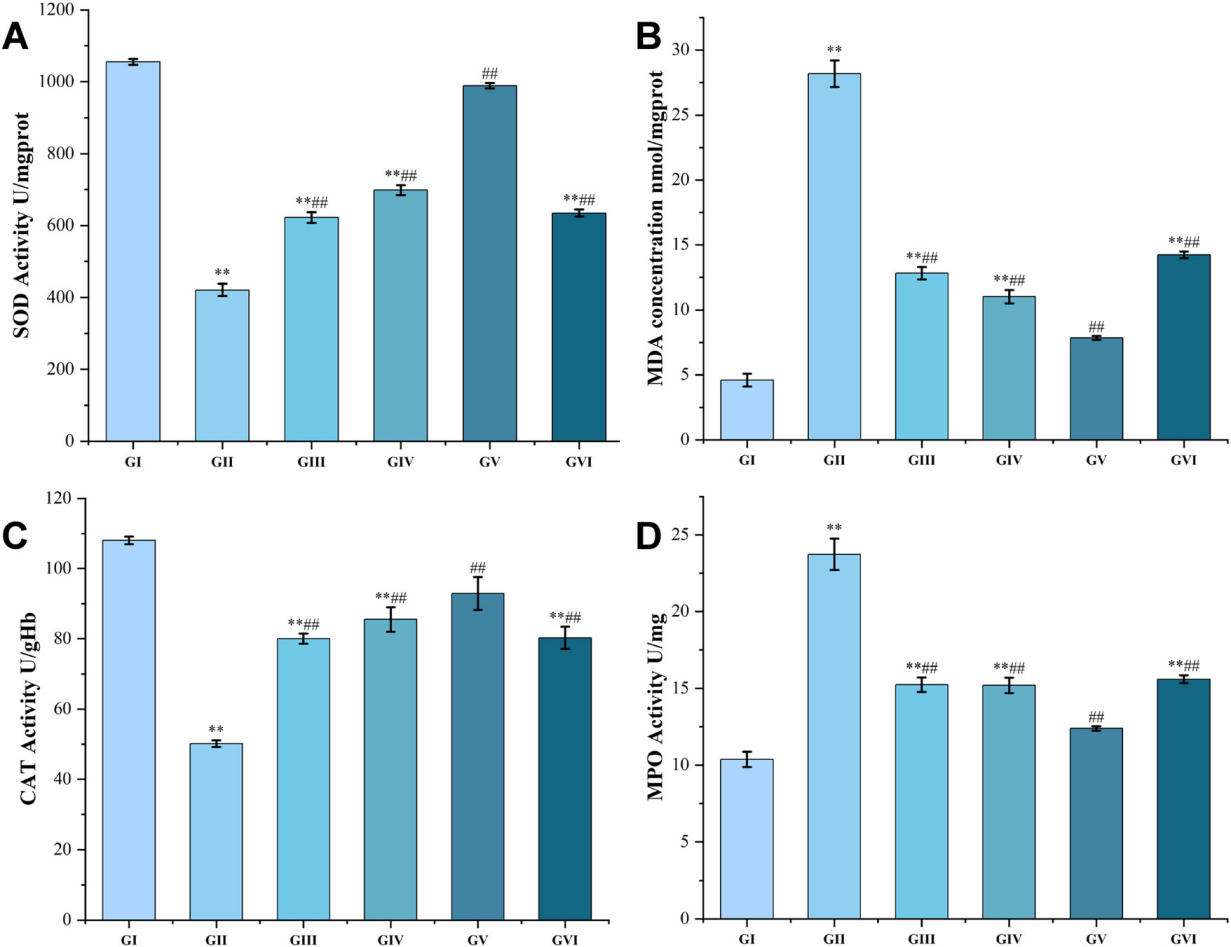
Effects of PPRHP on oxidative stress parameters in murine serum and gastric tissue ^**^represents *p* < 0.001 with the GI; ^##^represents *p* < 0.00*l* with the GII.

Oxidative stress imbalance is a key pathological feature of gastric ulcer that impedes wound healing. To assess whether PPRHP ameliorates this imbalance, we measured the activities of serum CAT and gastric tissue SOD, MPO, and MDA following treatment (Figure 4).

Compared with the normal group, the model group exhibited a marked disruption of redox homeostasis, characterized by significantly decreased activities of SOD and CAT (*p* < 0.001), together with elevated levels of MDA and MPO activity (*p* < 0.001). These results confirm that ethanol-induced gastric injury severely compromises the endogenous antioxidant defense system and promotes lipid peroxidation and neutrophil infiltration.

Administration of PPRHP, particularly at the medium dose, effectively counteracted these alterations. All PPRHP-treated groups and the omeprazole group showed significant restoration of SOD and CAT activities (*p* < 0.001), accompanied by a pronounced reduction in MDA content and MPO activity (*p* < 0.001). However, the extent of improvement varied with dose. Although the low-dose group exhibited increased SOD and CAT activities and decreased MDA and MPO levels (*p* < 0.005), its effect was less pronounced than that of the medium-dose group. Similarly, the high-dose group demonstrated only moderate improvements in these oxidative stress markers (*p* < 0.005), with efficacy also inferior to that of the medium-dose group.

These findings suggest that PPRHP, especially at 400 mg/kg, effectively restores oxidative-antioxidant balance in ethanol-induced gastric ulcers, primarily by enhancing antioxidant enzyme activities and suppressing lipid peroxidation and neutrophil activation.

## 4 Discussion

A methodological consideration of this study is that the standard acid hydrolysis used for amino acid analysis does not differentiate between aspartic acid and asparagine, or between glutamic acid and glutamine, and may lead to underestimation of sulfur-containing amino acids such as cysteine. More accurate quantification of these specific residues in the future would require advanced analytical techniques.

Furthermore, while the colorimetric assay employed here offers a reliable comparative evaluation of metal chelating activity, more absolute quantification of chelation capacity could be obtained in subsequent studies using methods such as atomic absorption spectroscopy or inductively coupled plasma mass spectrometry.

Alcohol-induced gastric ulcer represents a major ethanol-related digestive disorder, strongly linked to oxidative stress imbalance and compromised barrier function in the gastric mucosa [23]. The integrity of gastric epithelial cells, which undergo rapid turnover, is essential for maintaining gastric homeostasis. Impairment in their self-repair capacity can precipitate ulcerative pathologies. Current clinical management of alcohol-induced gastric ulcers relies primarily on acid-suppressive agents such as proton pump inhibitors and H_2_ receptor antagonists. While these drugs alleviate symptoms by reducing gastric acid secretion, they do not directly counteract oxidative mucosal injury or restore barrier function [24], 25]. In contrast, PPRHP – a bioactive peptide preparation – offers high specificity, biocompatibility, and low toxicity. Its multimodal mechanism, encompassing antioxidant and anti-inflammatory activities, enables direct mucosal protection, thereby addressing a critical gap in conventional therapies [26].

To evaluate the protective efficacy of PPRHP, an ethanol-induced gastric ulcer model was employed. Ethanol disrupts gastric mucosal homeostasis through dual pathogenic pathways. First, it directly impairs the mucus-bicarbonate barrier, promoting H^+^ back-diffusion and inducing mitochondrial electron transport chain uncoupling. This leads to a surge in mitochondrial ROS and highly toxic hydroxyl radicals, which initiate lipid peroxidation of unsaturated fatty acids in cell membranes and generate end products such as MDA [27]–29]. Second, ethanol suppresses the activity of endogenous antioxidant enzymes, including superoxide dismutase and catalase, thereby weakening the clearance of O_2_^−^ and H_2_O_2_. Concurrently, it activates MPO-mediated oxidative toxicity in neutrophils, resulting in ROS accumulation and lipid peroxidation in mucosal tissues. These processes form a self-amplifying cycle of “oxidative damage–barrier collapse” [30].

Oxidative stress and physical barrier damage synergistically exacerbate gastric mucosal injury. ROS directly oxidize tight junction proteins, disrupting intercellular junctions and increasing mucosal permeability. Meanwhile, hypochlorous acid generated by MPO degrades extracellular matrix components and promotes lipoprotein oxidation, further compromising membrane fluidity in epithelial cells [31], 32]. These processes collectively weaken gastric mucosal defense, where suppression of endogenous antioxidants and accumulation of oxidative mediators act as core drivers of pathology.

As a central mechanism in alcohol-induced ulcer formation, oxidative stress disrupts the redox balance via excessive free radical production, leading to lipid peroxidation, protein denaturation, and DNA damage [33], 34]. Previous studies support this view: Wang et al. [35] reported that exogenous antioxidants reduced gastric MDA levels by activating SOD and CAT, with SOD enhancement correlating dose-dependently with ulcer mitigation. Liu et al. [36] demonstrated that MPO inhibition attenuates neutrophil-derived ROS bursts and preserves mucosal integrity. Zhao et al. [37] observed that Nrf2-deficient mice showed diminished SOD and CAT activity and more severe ethanol-induced damage, whereas Wang et al. [38] found that Nrf2 activation upregulates antioxidant enzymes, suppresses lipid peroxidation, and promotes mucosal repair. Additionally, suppressed MPO activity further indicates the potential of bioactive peptides to alleviate neutrophil-mediated oxidative injury [39].

In line with these findings, the present study confirms that PPRHP possesses a rich amino acid profile and exhibits potent *in vitro* antioxidant properties. It demonstrated effective scavenging of ABTS and hydroxyl radicals, hydrogen peroxide decomposition, and copper ion chelation, likely attributable to redox-active residues within its peptide sequences.

Our findings align with a growing corpus of literature supporting the antioxidant and gastroprotective properties of protein hydrolysates from diverse sources. For example, bioactive peptides have been extensively documented to exhibit potent free radical-scavenging capacity and protective efficacy against ethanol-induced gastric ulcers in animal models [40], 41]. The underlying mechanisms frequently involve the upregulation of endogenous antioxidant enzymes, suppression of lipid peroxidation, and inhibition of pro-inflammatory pathways. The present investigation into PPRHP not only corroborates these established tenets but also underscores the considerable potential of *P. pastoris* fermentation residue as a novel and sustainable source of such bioactive peptides, thereby broadening the spectrum of available antioxidant hydrolysates.

The results of this study confirmed that ethanol induction elicited significant gastric ulceration and cellular necrosis in the model group, whereas animals treated with PPRHP (at low, medium, and high doses) or omeprazole displayed only minor white-spotted lesions and limited hemorrhage, along with markedly reduced mucosal damage area. Biochemically, ethanol challenge notably elevated MDA and MPO levels in gastric tissue, while depressing SOD and serum CAT activities. PPRHP administration reversed these changes, restoring SOD and CAT activities and suppressing MDA and MPO levels. Importantly, the medium-dose PPRHP group exhibited the most significant upregulation of SOD and CAT, aligning with previous reports that exogenous antioxidants can potentiate the endogenous enzymatic defense system [42]. These results suggest that the gastroprotective effect of PPRHP is mediated through scavenging free radicals, attenuating lipid peroxidation, and mitigating inflammatory activation.

Histopathological evaluation further demonstrated that medium-dose PPRHP alleviated epithelial cell exfoliation, mucosal congestion, and glandular structural disorganization, accompanied by reduced edema and hemorrhage. Consistent with morphological improvement, this group also showed the highest ulcer inhibition rate and the smallest ulcer area among all PPRHP-treated groups. The restorative effect of PPRHP on gastric mucosa is likely attributable to its dual antioxidant and anti-inflammatory properties. By rebalancing oxidative stress and suppressing inflammatory signaling, PPRHP helps preserve mucosal structural integrity and mitochondrial function, thereby attenuating ethanol-induced gastric damage.

The observed superior efficacy of the medium dose over the high dose across multiple parameters reflects a “bell-shaped” or biphasic dose-response, a recognized phenomenon in pharmacology and nutraceutical research, particularly with bioactive peptides and antioxidants. This pattern may be explained by two non-mutually exclusive mechanisms: first, saturation of intestinal peptide transporters or cellular receptors at high concentrations may limit bioavailability or signaling efficacy; second, certain antioxidant components in PPRHP may exhibit pro-oxidant activity under high-dose conditions, inducing mild oxidative stress that partially counteracts the protective effects observed at moderate doses.

While this study confirms the efficacy of PPRHP in an acute ethanol-induced gastric injury model, several limitations should be acknowledged. The acute model primarily reflects immediate oxidative and inflammatory damage from a single ethanol exposure and may not fully replicate the complex pathogenesis of chronic ulcers in humans, which often involve sustained inflammation, mucosal atrophy, *Helicobacter pylori* infection, and systemic factors. Nevertheless, the core mechanisms identified – enhancement of antioxidant defenses (SOD, CAT) and reduction of lipid peroxidation (MDA) and neutrophil infiltration (MPO) – are fundamental to mucosal repair and relevant to early alcohol-related damage. These findings provide a proof-of-concept for PPRHP’s potential, though future studies using chronic ethanol exposure or other established models (e.g., indomethacin-induced, stress-induced) are needed to assess its long-term therapeutic applicability.

It is also important to note that while key oxidative stress markers were quantified, deeper mechanistic insights – such as those obtainable through Western blot or qPCR analysis of related pathways – would strengthen the biological interpretation. Such approaches should be incorporated in subsequent investigations.

The dose selection (200, 400, and 800 mg/kg) was based on conventional practices in peptide studies and preliminary experiments, aiming to assess a broad efficacy range and dose-response behavior. The results validate both the safety and efficacy of this dosing strategy, with the most pronounced effects observed at 400 mg/kg. The ethanol-induced model was selected for its direct relevance to alcohol-related gastric injury, enabling focused evaluation of PPRHP’s antioxidant and barrier-protective properties under controlled oxidative challenge conditions.

Finally, it should be noted that this study focuses on the bioactivity of the total hydrolyzed peptide fraction. While the observed effects are likely primarily attributable to the peptide components, as suggested by their amino acid profile and known bioactive properties, we acknowledge that the potential contribution of minor non-peptide constituents (e.g., phenolic compounds or carotenoids) to the overall antioxidant activity was not quantified. Future work involving further purification of PPRHP and specific analysis of these non-peptide antioxidants would be valuable to conclusively establish the primary active principles.

## 5 Conclusions

This study demonstrates that PPRHP, derived from *P. pastoris* residue, is rich in amino acids, indicating high nutritional value. Furthermore, it exhibits significant *in vitro* and *in vivo* antioxidant and anti-inflammatory activities. These functional properties are likely attributable to the presence of specific reactive amino acid residues (such as histidine and tyrosine) within the peptide sequences, which confer free radical scavenging and metal ion chelation capacities. In a mouse model of ethanol-induced gastric injury, PPRHP exerted notable gastroprotective effects in a dose-dependent manner, primarily through mechanisms involving the scavenging of free radicals, inhibition of lipid peroxidation, and restoration of oxidative-antioxidative balance. These findings support the potential of PPRHP as a natural agent against alcohol-induced gastric damage and provide a scientific basis for the high-value utilization of *P. pastoris* fermentation by-products. However, this study is limited to an acute injury model, and the precise molecular mechanisms remain to be fully elucidated. Future work should focus on identifying the active peptide sequences, validating efficacy in chronic models, and exploring relevant signaling pathways to facilitate the translation of PPRHP into a clinically applicable nutraceutical.
